# Smart pH/Near-Infrared Light-Responsive Carboxymethyl Chitosan/Sodium Alginate/MXene Hydrogel Beads for Targeted Tea Polyphenols Delivery

**DOI:** 10.3390/gels11121009

**Published:** 2025-12-16

**Authors:** Kun Fang, Pei Li, Hanbing Wang, Xiangrui Huang, Yihan Li, Bo Luo

**Affiliations:** 1 Dabie Mountain Laboratory, College of Tea and Food Science, Xinyang Normal University, Xinyang 464000, China; 2 Henan Key Laboratory of Tea Plant Biology, Xinyang 464000, China; 3 Huaihe Campus Administrative Committee, Xinyang Normal University, Xinyang 464000, China

**Keywords:** smart hydrogels, tea polyphenols, sustained release

## Abstract

Tea polyphenols (TP) offer health benefits, but their stability is compromised by sensitivity to environmental factors, limiting their applications. Developing stimulus-responsive delivery systems that precisely control TP release is essential. This study prepared novel hydrogel beads encompassing carboxymethyl chitosan (CMC), sodium alginate (SA), and MXene (Ti_3_C_2_T_x_) using a blending method for the sustained release of TP. After being exposed to 808 nm near-infrared (NIR) radiation, the beads demonstrated excellent stability in simulated gastric conditions, resulting from the pH-dependent solubilization, facilitating controlled TP release under simulated intestinal conditions. The drug release kinetics conformed to the Ritger–Peppas model. Notably, CMC-SA-MXene@TP exhibited strong antioxidant activity and antimicrobial properties, effectively inhibiting the growth of *S. aureus (ATCC 6538)* and *E. coli (ATCC 25922)*. Additionally, according to in vitro cellular assays, they exhibited good biocompatibility with normal liver cells (HL-7702) and could effectively inhibit hepatocellular carcinoma cells (HepG2). These hydrogel beads, featuring excellent pH and NIR responsiveness, biocompatibility, drug loading efficiency, antioxidant capability, and antibacterial activity, represent promising candidates for advanced wound dressings or oral drug delivery systems for modulating intestinal flora.

## 1. Introduction

Consumers have increasingly recognized the health-promoting effects of bioactive substances, making these components popular in recent trends. Tea polyphenols (TP) are important bioactive components derived from tea, widely applied in food and biomedical industries due to their potent antioxidant activities and health benefits [1,2]. TP prevents chronic diseases, suppresses inflammation, controls blood lipid and glucose levels, protects the nervous system, enhances beneficial microbial populations, and maintains intestinal microbial balance, hence contributing to human health [3]. However, instability and low bioavailability remain major challenges that limit TP application [4]. Previous studies demonstrated that TP degrades easily in acidic conditions [5], whereas alkaline environments facilitate its absorption and utilization [6]. Thus, suitable strategies are necessary to protect TP from degradation and improve utilization. Inspired by precisely targeted drug delivery strategies in biomedicine, developing targeted TP delivery systems is a promising approach. TP has been delivered using various systems, including emulsions [7], microcapsules [8], nanoparticles [9], and liposomes [10]. However, each system has inherent limitations. For instance, emulsions enhance solubility but typically possess low mechanical strength and stability, complex production processes, sophisticated emulsification requirements, and high costs [11]. Nanoparticles allow precise size and shape control but may aggregate and disrupt cell membrane permeability, posing potential risks [12]. Hydrogel beads, however, offer notable advantages over these systems due to their biocompatibility, hydrophilicity, and ease of storage and transport [13].

The materials selected for hydrogel bead construction directly determine the delivery system’s effectiveness. Compared to synthetic polymers (e.g., polyester, polyvinyl alcohol, polyacrylic acid), natural polysaccharides exhibit superior stability, biodegradability, modifiability, and biocompatibility, hence becoming appropriate candidates for constructing delivery systems [14,15,16]. SA, a copolymer encompassing (1 → 4)-linked α-L-guluronic and β-D-mannuronic residues, varies in sequence by the source organism or tissue [17]. SA demonstrates physicochemical flexibility, cross-linking capability, and pH sensitivity [18]. To be specific, SA forms gels via coordination between carboxylate groups and multivalent cations such as Ca^2+^, Zn^2+^, Cu^2+^, Sr^2+^, Ba^2+^, and Al^3+^, resulting in a distinct “egg-box” structure [19]. However, SA chains contract under acidic conditions and expand in alkaline environments, leading to low mechanical strength, limited toughness, poor structural stability, and uncontrolled release of encapsulated substances [20]. Thus, combining SA with another polysaccharide can mitigate these limitations [21].

CMC, a derivative of chitosan, offers high solubility, viscosity, low toxicity, and good biocompatibility [22]. Enriched with carboxyl and amine groups, CMC forms non-covalent complexes with SA, providing structural stability while maintaining reversibility and flexibility [23]. Furthermore, CMC exhibits antibacterial, antifungal, and antioxidant activities, and thus can be broadly applied in wound healing, tissue engineering, cancer therapy, biosensors, bioimaging, etc. [24]. Additionally, CMC finds applications in food industries as smart packaging materials, food additives, bioactive ingredient carriers, and food sensors [25].

Nevertheless, natural polymer-based delivery systems lack sensitivity to light, electrical signals, and other physical stimuli. Identifying suitable fillers to address this issue remains challenging. MXenes, a new type of versatile two-dimensional nanosheets (M_n+1_X_n_T_x_, where n = 1–3, M is transition metals, X is carbon or nitrogen, and T_x_ is surface terminations of -OH, -F, or -O), have overcome these limitations. MXenes have been well studied, relying on the layered architecture, strong electrical conductivity, significant chemical stability, hydrophilicity, large surface-to-volume ratio, abundant functional groups, and biocompatibility [26]. Rakkan et al. [27] previously synthesized cellulose nanofiber-reinforced chitosan/poly (vinyl alcohol)/MXene (Ti_3_C_2_T_x_) membranes for controlled quercetin release in wound healing, where MXene enhanced quercetin adsorption and improved mechanical properties, such as strength, Young’s modulus, and toughness.

Against this backdrop, surface functionalization of MXenes with natural polymers like CMC and SA provides an effective bridge to construct delivery systems. In this study, MXene nanosheets were first synthesized through an etching approach. Subsequently, novel CMC-SA-MXene@TP hydrogel beads with spherical structures were developed via a cross-linking method, facilitating effective controlled TP release in response to pH and near-infrared (NIR) radiation. The biocompatibility and antibacterial properties of these smart hydrogel beads were evaluated. Overall, CMC-SA-MXene@TP hydrogel beads represent significant potential for advanced applications in polysaccharide/MXene-based active substance delivery technologies.

## 2. Results and Discussion

### 2.1. Characterization Analysis

**Figure 1:**
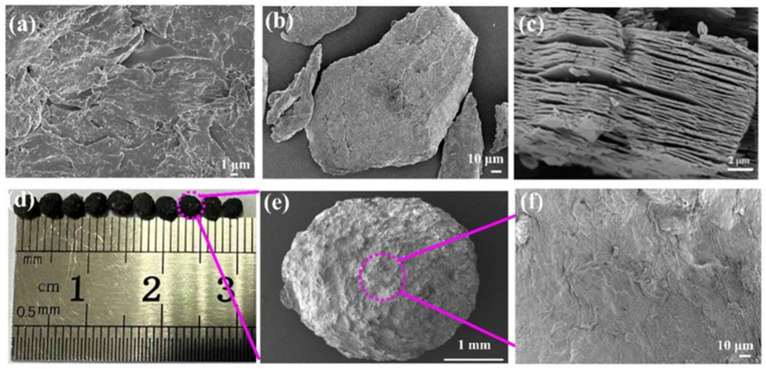
SEM images of (**a**) SA, (**b**) CMC, (**c**) MXene; digital photograph (**d**) and SEM images (**e**,**f**) of CMC-SA-MXene@TP hydrogel beads.

The SEM micrographs illustrate the samples’ morphology. Figure 1a shows SA with a smooth surface. Figure 1b demonstrates that CMC possesses an amorphous structure featuring a rough surface characterized by hollows and folds. Figure 1c reveals etched MXene with a distinct multilayer stacked “accordion-like” morphology, resulting from aluminum layers being selectively removed from Ti_3_AlC_2_ in the etching process [28]. The residual MXene (Ti_3_C_2_T_X_) layers stack densely or loosely via weak van der Waals interactions or binding through functional groups (-OH, -O, -F). In Figure 1d, the hydrogel beads had an average diameter ≈ 3.1 mm, providing adequate surface area for TP loading. In Figure 1e,f, the hydrogel beads exhibit a highly textured surface with obvious folds and wrinkles. This morphology could result from (1) interactions among CMC, SA, and MXene nanosheets, strengthening the cross-linking network within the hydrogel, and (2) structural shrinkage caused by water removal during freeze-drying, leading to surface wrinkling [29]. Despite structural modifications, hydrogel beads retained their original shape, hinting at excellent mechanical stability, which is advantageous for practical applications. These results correspond to the XRD findings, where only the strongest MXene diffraction peaks were observed in the XRD analysis data for the hydrogel beads, while the characteristic diffraction peaks of CMC and SA were not detected.

**Figure 2:**
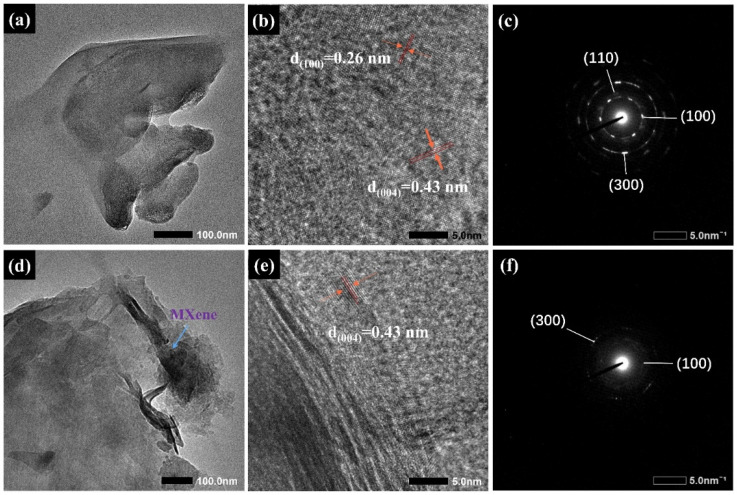
TEM, HR-TEM, and SAED images of MXene (**a**–**c**) and crushed CMC-SA-MXene@TP hydrogel beads (**d**–**f**).

The Ti_3_C_2_T_X_ nanosheets appear thin, transparent, and partially stacked in Figure 2a. In Figure 2b, Ti_3_C_2_T_X_ nanosheets exhibit obvious lattice fringes with spacings of 0.43 nm and 0.26 nm, targeting the (004) and (100) crystal planes, respectively [30]. SAED analysis (Figure 2c) reveals clear concentric diffraction rings resulting from the (100), (110), and (300) crystal planes, ascertaining that MXene’s intrinsic crystalline structure is preserved [31]. These results align with previous reports, further confirming the successful etching of Ti_3_C_2_T_X_ [32,33]. The TEM image (Figure 2d) of pulverized CMC-SA-MXene@TP beads displays unique MXene lamellar structures within the polymer matrix. Figure 2e demonstrates Ti_3_C_2_T_X_ crystal features being retained within the composite, particularly the 0.43 nm lattice fringes (004) plane. However, the corresponding SAED pattern (Figure 2f) appears blurrier, possibly because amorphous polymer components (CMC, SA) can be observed, yet still preserves diffraction ring information corresponding to (100) and (300) planes. Additionally, certain peaks have weakened or disappeared, indicating that after being encapsulated by CMCS/SA, the diffraction peaks of the material have been masked or their intensity significantly reduced.

**Figure 3:**
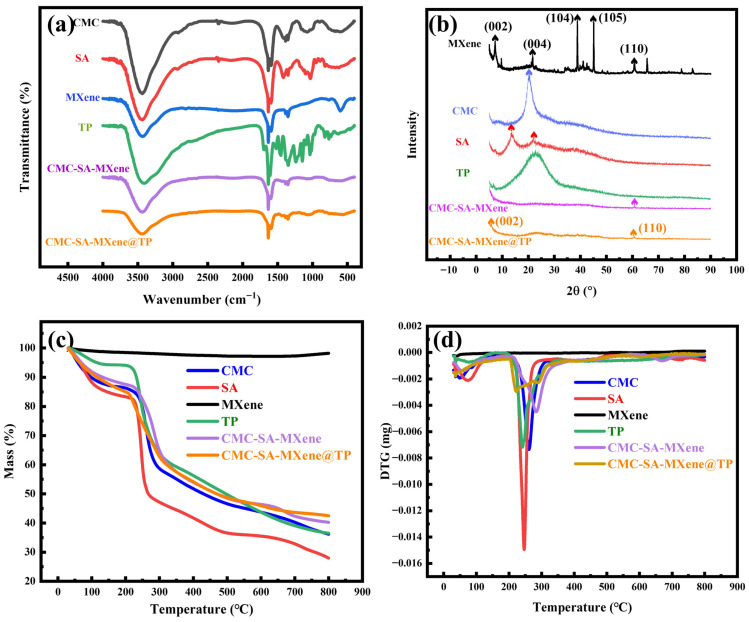
(**a**) FT-IR spectra, (**b**) XRD patterns, and (**c**,**d**) TG and DTG curves of CMC, SA, MXene, TP, CMC-SA-MXene, and CMC-SA-MXene@TP.

Figure 3a shows FTIR spectra of various samples, highlighting characteristic molecular vibrations. In the CMC spectrum, peaks at approximately 3300 cm^−1^ correspond to -OH and -NH stretching vibrations, 2866 cm^−1^ relates to C-H stretching, and the absorption near 1597 cm^−1^ corresponds to COO- stretching vibrations [34]. Characteristic absorption peaks of SA appear at 2930 cm^−1^ (C-H stretching), 1640 cm^−1^ and 1422 cm^−1^ (C-O-O vibrations), and 1090 cm^−1^ (C-O-C vibrations) [35]. The TP spectrum exhibits characteristic peaks at 3352–3295 cm^−1^, 3262 cm^−1^, 1691 cm^−1^, 1610 cm^−1^, 1446 cm^−1^, 1342 cm^−1^, 1215 cm^−1^, 1141 cm^−1^, and 1010 cm^−1^, representing O-H stretching vibrations, C-H vibrations, benzene ring C-C skeletal vibrations, O-H bending vibrations in phenolic hydroxyl groups, asymmetric and symmetric C-O-C ether bond stretching vibrations, and phenolic C-O stretching vibrations, respectively [36]. The MXene spectrum displays distinct bands at 3427 cm^−1^ and 1628 cm^−1^, assigned to surface hydroxyl groups, but the peak at 1097 cm^−1^ targets C-F bond stretching vibrations [37]. The composite CMC-SA-MXene retains all characteristic peaks from the CMC and SA components, exhibiting weak peaks in the 3500–3000 cm^−1^ range, likely originating from C–H bonds and hydroxyl functional groups within the hydrogel. Compared to SA and CMCS, the hydroxyl stretching vibration peak of CMC-SA-MXene red-shifted from 3415 cm^−1^ and 3417 cm^−1^ to 3408 cm^−1^. This suggests the presence of intermolecular hydrogen bonding interactions within the matrix [38]. Additionally, the distinctive C-F stretching vibration at 1093 cm^−1^ confirms MXene incorporation within the composite hydrogel matrix. CMC-SA-MXene@TP maintains the spectral features of CMC-SA-MXene and shows additional characteristic TP peaks at 1143 cm^−1^ and 1680 cm^−1^, indicating successful TP loading. All composites exhibit absorption bands around 3400 cm^−1^ (O-H stretching) with similar intensities. Nevertheless, the O-H stretching band in MXene exhibits weaker intensity and slight frequency shifts, likely caused by the partial dehydroxylation in the high-temperature processing. Moreover, the characteristic -OH absorption peaks of CMC-SA-MXene and CMC-SA-MXene@TP at 3290 cm^−1^ became weaker, suggesting possible hydrogen bonding and electrostatic interactions involving hydroxyl, amino, and carboxyl groups [39].

Figure 3b reveals characteristic peaks at 2θ = 9.5°, 19.6°, 39.6°, 42.3°, and 60.4°, assigned to the (002), (004), (104), (105), and (110) crystal planes, respectively [40]. Following etching treatment, the characteristic MAX phase peak at 40° disappears, while a prominent Ti_3_C_2_T_X_; peak emerges at 2θ = 8.5°, which confirms the successful MXene synthesis as previously described. The SA pattern exhibits 2 diffraction peaks at 2θ = 13.6° and 22.5°. Also, CMC and TP show broad diffraction peaks near 2θ = 20.8° and 22.6°, indicating their amorphous structures. In CMC-SA-MXene and CMC-SA-MXene@TP composites, SA peaks disappear, suggesting increased amorphous character. This structural transformation may result from the disruption of SA crystalline regions due to Ca^2+^-mediated crosslinking with CMC [41]. Furthermore, MXene’s characteristic diffraction peaks corresponding to (002) and (110) crystal planes are observed in CMC-SA-MXene and CMC-SA-MXene@TP, demonstrating that MXene is successfully integrated into the hydrogel matrix. 

In Figure 3c,d, MXene exhibited only a minor mass loss, corresponding to adsorbed water removal, without significant degradation up to 800 °C. The TGA and DTG curves in Figure 3c (30–800 °C) depict a comparable three-stage decomposition pattern. The first stage of mass loss (30–150 °C) appeared mainly because physically adsorbed water was evaporated [42]. The second stage (180–380 °C) involved polymeric component degradation, namely saccharide ring rupture, polymer chain fragmentation, glycosidic bond cleavage, and intra- and intermolecular interaction disruption [43]. The final decomposition stage was observed above 500 °C, during which residual organic components were oxidized, and carbonaceous residues were formed [44]. At the conclusion of degradation, the mass losses for CMC, SA, TP, CMC-SA-MXene, and CMC-SA-MXene@TP were 63.80%, 72.01%, 63.49%, 59.77%, and 57.46%, respectively, indicating that CMC-SA-MXene@TP had the highest thermal stability. Interestingly, hydrogel bead stability increased upon TP addition, likely due to interactions between TP and the CMC-SA-MXene matrix. The DTG curves (Figure 3d) identified maximum decomposition rates (T_max_) at 254.5 °C (CMC), 246.7 °C (SA), 242.2 °C (TP), 285.1 °C (CMC-SA-MXene), and 289.5 °C (CMC-SA-MXene@TP). Analysis of T_max_ revealed that adding CMC elevated SA’s decomposition temperature, as evidenced by the weaker polymer chain mobility in CMC-SA networks coupled with more rigid intermolecular structures; thus, higher thermal energy was required for decomposition [45]. Furthermore, including thermally stable MXene enhanced the thermal stability of CMC-SA-MXene and CMC-SA-MXene@TP compared to pure SA and CMC. As reported, MXenes, as conductive nanoparticles, might induce cross-linking between polymer chains through their surface hydrophilic groups. This enhanced intermolecular forces, significantly improving the mechanical properties of the copolymer gel—particularly in tensile strength and toughness—while also boosting its thermal stability [46].

### 2.2. TPC and Release Studies

As shown in Table 1, the TPC values for the respective hydrogel beads were 15.68%, 34.43%, and 37.02%, respectively. Additionally, TPC in the hydrogel beads increased with increasing TP content. CMC, SA, and MXene contain abundant hydroxyl groups (-OH), allowing interactions with TP molecules via hydrogen bonding. Therefore, TP molecules adsorb onto MXene nanosheets as well as onto CMC and SA surfaces. Many active sites on CMC-SA-MXene remain unoccupied at lower TP concentrations. With increasing TP concentration, these active sites gradually become saturated, resulting in increased loading capacity. Notably, at a TP addition of 0.75 g, the loading rate of CMC-SA-MXene@TP-3 only rose by 2.59%. This modest increase occurred because of the occupation of most active sites on CMC-SA-MXene by TP molecules, making it difficult to further enhance loading through increased TP concentration.

### 2.3. TP Release Studies

**Figure 4:**
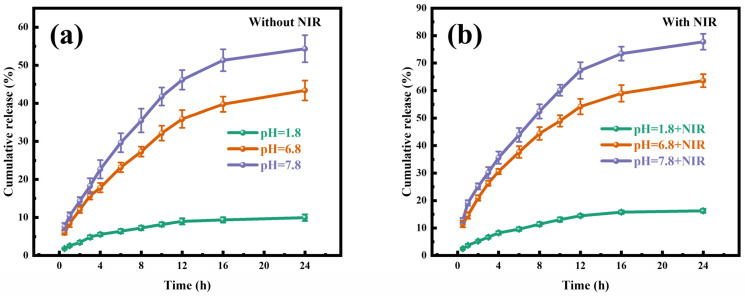
Cumulative drug release profiles of hydrogel beads in varying media, (**a**) with and (**b**) without NIR irradiation.

In Figure 4, cumulative TP release profiles closely depended on medium pH and NIR irradiation. After 24 h incubation without NIR irradiation in simulated gastric fluid (SGF, pH 1.8), the cumulative TP release was still less than 13%. In contrast, drug release was about 50% at pH 6.8 and 65% at pH 7.8. The minimal TP release was detected at acidic pH, resulting from the hydrogel matrices’ low solubility and limited swelling. The significantly higher release at pH 7.8 arose from the ionization of carboxyl groups in the polymers (CMC and SA) in weakly alkaline conditions. This brings in a loosened hydrogel network and increased solubilization capacity, facilitating TP dissolution. In the previous research, due to the abundance of hydrophilic groups on their surfaces, composite hydrogel beads exhibit strong water absorption capabilities. Under acidic conditions, carboxyl groups (-COOH) dominate, causing the hydrogel structure to contract through hydrogen bonding and coordination interactions, thereby reducing expansion. Conversely, in alkaline environments, -COOH dissociates into -COO^−^, enhancing electrostatic repulsion and accelerating the expansion rate. Accompanying the swelling process, rupture phenomena occur, which may ultimately lead to the destruction of the composite hydrogel beads’ three-dimensional structure [47]. Additionally, the initial TP release was fast within the first 4 h because surface-bound drug molecules readily detached from the gel surface. Sustained drug release was detected over 4–24 h. Under NIR irradiation, TP release significantly increased compared to standard conditions (NIR-free), resulting in approximately a 9% increase in cumulative release after 6 h. Moreover, NIR exposure facilitated more efficient TP release than passive diffusion alone. However, at pH 1.8, NIR irradiation contributed minimally to drug release. The enhanced TP release may be attributed to the photothermal effect generated by MXene nanosheets when hydrogel beads are exposed to laser irradiation. Additionally, the elevated temperature of the gel beads increases the structural motion of the CMCS/SA complex, accelerating hydrolysis and thereby promoting efficient TP release, which was similar to the findings reported by Chen et al. regarding the release of paclitaxel from HA-PLGA/MX NPs under NIR exposure [48]. Thus, dual-responsive (pH and NIR) CMC-SA-MXene@TP hydrogels provided effective TP preservation under acidic gastric conditions, meanwhile enabling controlled drug release across various physiological environments.

According to Table S1 and Figures S1 and S2 (Supporting Information), the Ritger–Peppas model demonstrated the highest correlation coefficient (R^2^ = 0.9817) at pH 7.8, compared to the zero-order (R^2^ = 0.9125), first-order (R^2^ = 0.9479), and Higuchi models (R^2^ = 0.9811). Thus, the Ritger–Peppas model best described experimental data, consistent with observations under NIR irradiation. The Ritger–Peppas framework adopts diffusion exponent values (n) to define the release mechanisms: n < 0.43 denotes predominantly Fickian diffusion; 0.43 < n < 0.85 denotes anomalous transport (combined Fickian diffusion and swelling), and n > 0.85 denotes primarily swelling-controlled release (case II transport) [49]. According to the analysis results of parameters in each model, TP release was primarily dominated by combined Fickian diffusion and swelling at pH 1.8, and it was governed by Fickian diffusion, swelling, and erosion at pH 6.8 and 7.8. The TP release mechanism from CMC-SA-MXene@TP hydrogel beads involved three stages: First, since the TP concentration within the gel microspheres is significantly higher than that in the medium, TP dissolution is primarily governed by diffusion. Initially, the polymer exhibits low water absorption, and TP movement is restricted by the microporous size limitations. Second, water molecules diffuse into the gel microspheres, enlarging their pore size and inducing microsphere swelling and relaxation, thereby promoting sustained drug release. Finally, sustained water permeation erodes the polymer matrix within the gel microspheres, leading to complete relaxation and full hydration, which further accelerates the dissolution of TP from the gel microspheres into the medium.

### 2.4. Antioxidant Activity Test

**Figure 5:**
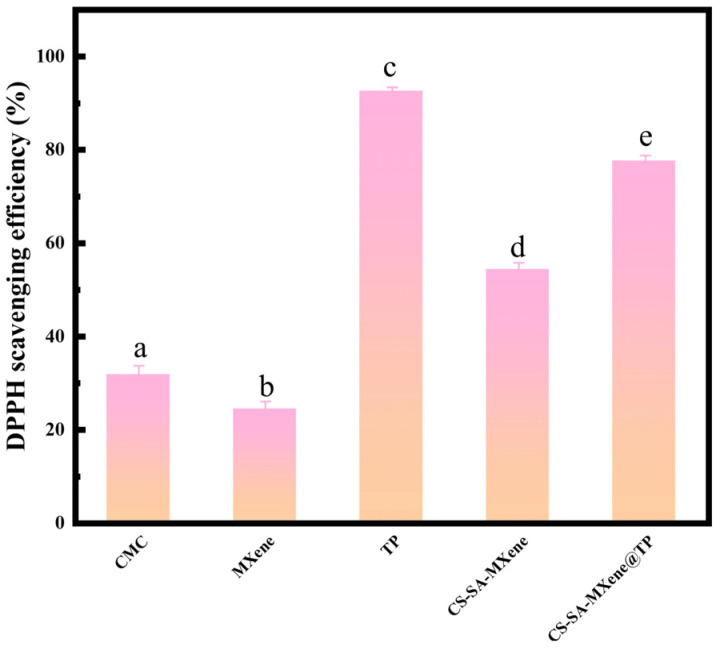
Samples’ DPPH radical scavenging activity. (Different superscript letters mean significant differences in the same column (*p* < 0.05)).

The DPPH assay is widely utilized to objectively assess the antioxidant capability by measuring free radical scavenging activities. According to Figure 5, CMC exhibited moderate antioxidant activity (31.71%), attributed to its carboxymethyl groups, which possess hydrophilic and negatively charged characteristics. These groups can capture radicals such as hydroxyl radicals and superoxide anions via hydrogen bonding or electrostatic interactions, thus directly exerting antioxidant effects possibly related to molecular weight [50]. MXene demonstrated a DPPH scavenging activity of 24.23%, primarily affected by the intrinsic reducing properties of Ti_3_C_2_T_x_ instead of surface functional groups. Upon interaction with DPPH radicals, Ti–C bonds in Ti_3_C_2_T_x_ initially break, forming layered carbon atoms and isolated Ti ions. Subsequently, the surface defects of layered carbon atoms adsorb DPPH radicals and oxygen-containing functional groups from water [51]. The highest antioxidant activity was observed in TP (92.61%), mainly attributable to natural phenolic compounds. TP molecules neutralize free radicals by donating small, highly reactive hydrogen protons, thus terminating radical chain reactions [52]. Due to antioxidant components (CMC, MXene, TP), CMC-SA-MXene and CMC-SA-MXene@TP achieved DPPH scavenging rates of 54.23% and 77.56%, respectively.

### 2.5. Antibacterial Activity Analysis 

**Figure 6:**
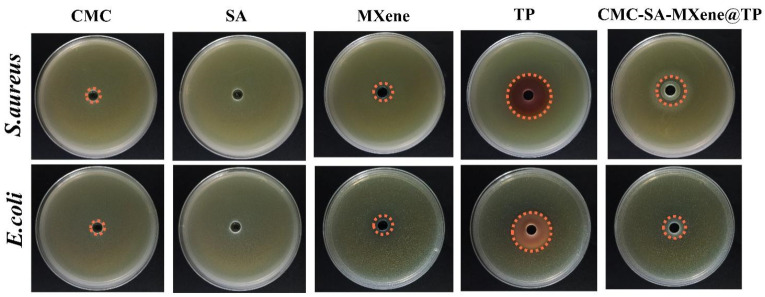
Samples’ antibacterial activity against *E. coli* and *S. aureus* (Initial zone diameter: 6 mm).

This study focused on evaluating samples’ antibacterial activity against *E. coli* and *S. aureus* (Figure 6). Table 2 provides a detailed description of the zone sizes for *Escherichia coli* and *Staphylococcus aureus* across different samples. CMC reportedly has antibacterial properties against various microorganisms, represented by *E. coli*, *S. aureus*, and *B. subtilis* [24]. For instance, N-CMC shows greater bactericidal activity compared to chitosan [53], whereas for O-CMC, the antimicrobial effectiveness is related to its deacetylation degree, concentration, molecular weight, and solution pH [54]. However, CMC demonstrated limited antibacterial activity, possibly due to reduced NH^3+^ groups and a high substitution degree. The molecular structure of SA contains no direct antibacterial groups; thus, it exhibited negligible bacteriostatic effects. MXene displayed moderate bacteriostatic activity, attributed primarily to its capability of disrupting bacterial cell membranes, inducing cell damage and death [55]. The antibacterial activity of TP against *S. aureus* (31.33 mm) was higher than that against *E. coli* (26.58 mm), potentially due to differences in bacterial cell wall structure. The single-layer cell wall of *S. aureus* was more susceptible to polyphenol-induced damage, which enhanced membrane permeability and caused leakage of intracellular substances, thereby disrupting bacterial metabolism and inhibiting growth [56]. CMC-SA-MXene@TP exhibited significant antibacterial activity resulting from the combined antibacterial properties of CMC, MXene, and TP, with inhibition zone diameters of 19.29 mm for *S. aureus* and 15.31 mm for *E. coli*. The zone of inhibition results indicated that the antibacterial zone formed by CMC-SA-MXene@TP was smaller than that of free TP, suggesting restricted diffusion and reduced apparent antibacterial efficacy. This was attributed to the encapsulation technology limiting the immediate release of TP, thereby diminishing its short-term antibacterial diffusion effect in the culture medium.

### 2.6. Cell Cytocompatibility and Toxicity Test

**Figure 7:**
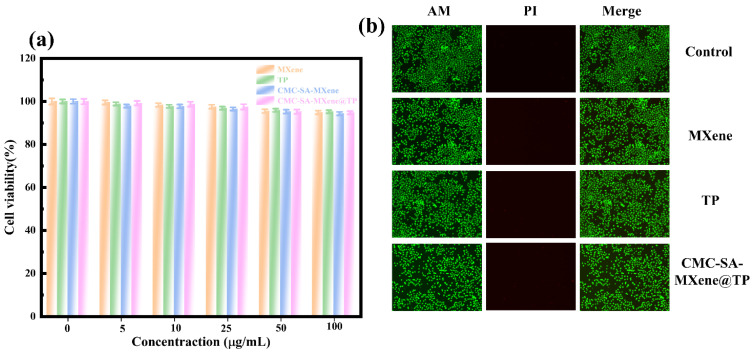
(**a**) In vitro cytocompatibility against HL-7702 cell lines; (**b**) Fluorescent images of HL-7702 cells after treated with different samples and followed with Cal-cein-AM and PI staining.

MTT assays evaluated the cytotoxic effects of TP, MXene, CMC-SA-MXene, and CMC-SA-MXene@TP on HL-7702 hepatocytes. Figure 7a shows that the survival rate of HL-7702 cells decreases slightly with increasing concentration, indicating dose-related cytotoxicity. Conversely, cell viability remained mostly above 90% after exposure to all samples at concentrations below 100 μg/mL, suggesting minimal cytotoxicity. Subsequently, complementary live/dead fluorescence assays visualized cellular responses using confocal microscopy. Calcein-AM is a membrane-permeable dye, and its activation relies on intracellular esterases. It emits green fluorescence in live cells. Propidium iodide (PI) selectively penetrates damaged cell membranes, generating red fluorescence in dead cells [57]. In Figure 7b, nearly all cells appear green, indicating that none of the materials induced cell apoptosis. Taken together, engineered CMC-SA-MXene@TP hydrogel beads demonstrate low cytotoxicity and limited toxicity at higher dosages, enabling them to be effective biocompatible drug delivery carriers.

**Figure 8:**
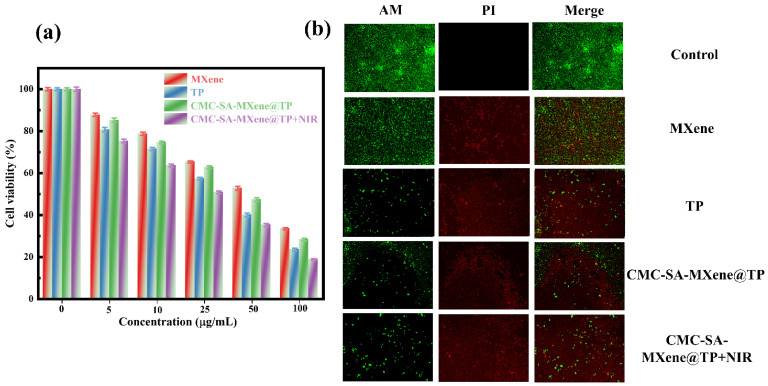
(**a**) In vitro cytocompatibility against HePG2 cell lines; (**b**) Fluorescent images of HepG2 cells after treated with different samples and followed with Calcein-AM and PI staining.

In vitro, antiproliferative efficacy critically assesses therapeutic potential for cancer-targeting drug delivery systems. Exposure to free TP and CMC-SA-MXene@TP at increasing concentrations demonstrated concentration-dependent cytotoxic effects on HepG2 hepatocarcinoma cells in Figure 8a. The IC_50_ value, which denotes the concentration that inhibits 50% of cell proliferation [58], was 57.02 μg/mL (MXene), 35.43 μg/mL (TP), 45.61 μg/mL (CMC-SA-MXene@TP), and 26.51 μg/mL (CMC-SA-MXene@TP+NIR). MXene produces biological activity in living cells by generating reactive oxygen species (ROS) [59]. Figure 8b demonstrates that all treatment groups, except the control, primarily exhibited red fluorescence, indicating apoptosis. Hence, TP, CMC-SA-MXene@TP, and CMC-SA-MXene@TP+NIR triggered apoptosis. Notably, NIR irradiation (808 nm laser) accelerated apoptotic cell death in the CMC-SA-MXene@TP group. These findings illustrate the enhanced cytotoxic efficacy of CMC-SA-MXene@TP when combined with 808 nm NIR irradiation, emphasizing that it can serve as a responsive drug delivery platform in cancer treatment. 

## 3. Conclusions

The present study described the preparation of a multi-responsive (pH and NIR) CMC-SA-MXene@TP hydrogel bead delivery system. The hydrogel beads demonstrated pH-sensitive release profiles in the digestion process, enabling slow TP release in the gastric fluid to prevent rapid degradation and accelerated release in the intestinal fluid to strengthen bioaccessibility. This controlled release resulted from the structural properties of SA and CMC, which provided pH responsiveness, as well as MXene, which introduced photothermal responsiveness. Pharmacokinetic analysis demonstrated a multimodal TP release mechanism involving diffusion, swelling, and erosion, fitting primarily to the Ritger–Peppas model. Additionally, CMC-SA-MXene@TP exhibited enhanced antioxidant activity (77.56%) and antibacterial efficacy against *E. coli* and *S. aureus*. In cellular assays, the hydrogel beads exhibited outstanding biocompatibility, and TP was effectively released, significantly inducing HCT116 cancer cell death under NIR radiation or alkaline conditions. In summary, the cost-effective raw materials and environmentally friendly synthesis of these multifunctional hydrogels offer notable advantages, such as stimuli-responsive carriers for developing polysaccharide-based and TP-loaded food-grade delivery platforms and gel-based functional foods and biomedical fields.

## 4. Materials and Methods

### 4.1. Materials

The MAX phase (Ti_3_AlC_2_), lithium fluoride (LiF, 99%), CMC (deacetylation degree ≥ 95%, viscosity 100–200 mPa-s), TP (purity 97%), gallic acid (GA, purity 99%), SA (MW = 4.8 × 10^6^ ± 1.8 × 10^5^ Da; M/G ratio: 0.59/1), and 2-diphenyl-1-picrylhydrazyl (DPPH, MW = 394.32, purity 97%) were purchased from Macklin Biochemical. (Shanghai, China). The cell viability reagent MTT, DMEM (high glucose), and PBS buffer solutions (pH = 1.8, 6.8, and 7.8) were obtained from Yuanye Biotechnology Co. (Shanghai, China). Gram-positive *Staphylococcus aureus* (*S. aureus*, ATCC 6538) and Gram-negative *Escherichia coli* (*E. coli*, ATCC 25922) were sourced from the American Type Culture Collection (ATCC). All other chemical reagents used (Sinopharm Chemical Reagent, Shanghai, China) were of analytical grade. Milli-Q ultrapure water prepared in the laboratory was used across the experiments.

### 4.2. Fabrication of MXene Nanosheets

MXene nanosheets were prepared using a LiF/HCl-mediated etching method [60]. At first, 1.16 g LiF and 20 mL of hydrochloric acid (9 M) were put in a Teflon container and underwent constant magnetic stirring for full mixing. Then, the acidic solution was added with 1 g Ti_3_AlC_2_ powder and allowed for 48 h of etching at 35 °C. After the reaction, the suspension underwent centrifugation, with the precipitate repeatedly washed in deionized (DI) water until the pH exceeded 6. The obtained multilayer MXene structures dispersed in 100 mL of distilled water were subjected to 1 h of sonication in a nitrogen atmosphere. The resultant dispersion underwent centrifugation (4000 rpm, 1 h), and the collected supernatant underwent freeze-drying to yield single/few-layer MXene nanosheets.

### 4.3. Construction of CMC-SA-MXene@TP Hydrogel Beads

**Figure 9:**
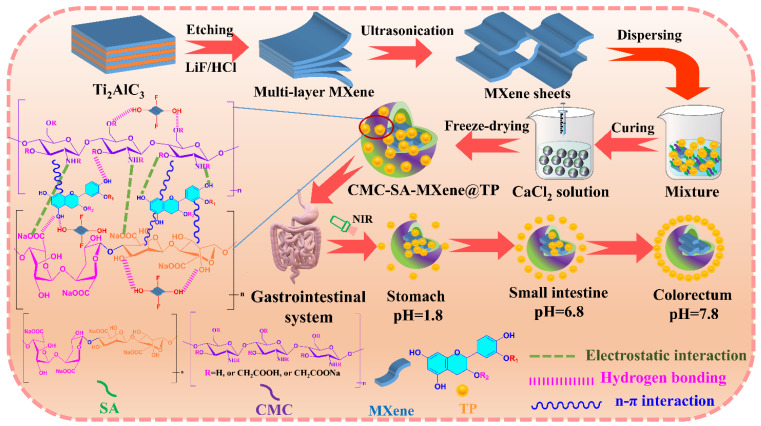
Schematic plot illustrating the preparation and release process of CMC-SA-MXene@TP hydrogel beads.

The preparation of hydrogel beads was modified as per our previous method [61]. Briefly, 1.0 g CMC, 1.5 g SA, 0.5 g MXene, and 0, 0.25, 0.5, and 0.75 g TP were dissolved in distilled water (100 mL) and magnetically stirred in a water bath (60 °C, 400 rpm, 4 h). The mixture underwent sonication to eliminate trapped air bubbles. After cooling, the solution was dripped into 4% (*w*/*v*) calcium chloride solution and incubated for 2 days. The filtered mature hydrogel beads underwent a full wash in distilled water to eliminate the residual calcium ions and TP. At last, the purified beads underwent lyophilization and were preserved under suitable conditions. The schematic diagram for preparing CMC-SA-MXene@TP beads is shown in Figure 9. For clarity, beads with varying TP contents were labeled CMC-SA-MXene@TP-0, CMC-SA-MXene@TP-1, CMC-SA-MXene@TP-2, and CMC-SA-MXene@TP-3.

### 4.4. Characterizations

Surface morphology and elemental composition were examined with SEM (Sirion 200, FEI, USA) (accelerating voltage: 10 kV). TEM (Tecnai G2 F20, FEI, Hillsboro, OR, USA) evaluated the morphological and dimensional properties. An FT-IR spectrophotometer (Nicolet iS10, Nicolet Instrument, Madison, WI, USA) was adopted to record the FT-IR spectra. The crystallographic structure was examined by XRD (Rigaku Ultima IV, Rigaku, Tokyo, Japan) (accelerating voltage: 40 kV; current: 30 mA), over a scanning range of 5–80° (2θ) at 5° min^−1^. Thermal decomposition behavior was determined with TGA/DTG (Netzsch 449 F5, Selb, Germany) under a nitrogen flow (20 mL/min) at 10 s °C/min. 5–10 mg samples were heated from 30 to 800 °C in alumina crucibles. CLSM (Leica TCMC SP8 DIVE system, Wetzlar, Germany) served for visualizing the fluorescence distribution. 

### 4.5. Determination of TP Content (TPC)

The study measured the TPC in hydrogel beads by the Folin–Ciocalteu method, with slight modifications from Benlloch-Tinoco et al. [62], first dissolving 60 mg of the sample in 20 mL PBS buffer solution (pH = 7.8) at ambient temperature with constant stirring for a complete dissolution. Next, the mixture of 1 mL of hydrogel bead extract and 1 mL of Folin–Ciocalteu reagent underwent 5 min of incubation in the dark, followed by the addition of 1 mL of 10% (*w*/*v*) Na_2_CO_3_ solution. The resulting solution was mixed with distilled water to a total volume of 25 mL. Following 2 h of incubation in the dark, a UV spectrophotometer (1000 Series, Talbot Scientific, Salisbury, UK) was used for measuring the absorbance at 765 nm. From a gallic acid calibration curve (A = 0.4898C + 0.0155, R^2^ = 0.9993), the TPC of the hydrogel beads was expressed as mg gallic acid equivalent (GAE)/g hydrogel beads. TPC was calculated using Equation (1):

(1) $$\mathrm{LC}(\%)=\frac{W_{TP}}{W_{S}}\times 100\%$$

where *W_TP_* is the mass of TP in CMC-SA-MXene@TP (g), and *W_S_* is the total mass of CMC-SA-MXene@TP (g). 

### 4.6. TP Release Under pH and NIR Stimuli

Drug release experiments involved placing freeze-dried CMC-SA-MXene@TP hydrogel beads (0.2 g) in a semipermeable membrane (MW cut-off: 3000 Da). Membranes were immersed in 30 mL sterile simulated digestive media without enzymatic components at pH values of 1.8, 6.8, and 7.8 [63]. The vessels underwent 24 h of incubation at 37 ± 1 °C and 200 rpm. At designated intervals, we conducted spectrophotometric analysis on aliquots (3.0 mL) at 765 nm and added equal volumes of fresh buffer at the corresponding pH to keep a constant medium volume. Additionally, the TP release variation under NIR photothermal stimulation (808 nm, 1.5 W/cm^2^) was examined using a similar procedure. At various intervals (30, 60, 120, 180, 240, 360, 480, 600, 720, 960, and 1440 min), hydrogel beads in buffer solutions (pH = 1.8, 6.8, 7.8) were exposed to NIR irradiation for 5 min. Released TP was quantified using the previously established calibration curve. Equation (2) is formulated to calculate the cumulative TP release percentage:

(2) $$R=\frac{V_{1}\times C_{n}+V_{2}\times \Sigma C_{n-1}}{w_{0}}\times 100\%$$

where *R* denotes the cumulative percentage of released TP, *V*_1_ is the total medium volume, *V*_2_ indicates sampling volume at each interval, C_n_ and C_n−1_ represent TP concentrations (mg/mL) at the nth and previous sampling intervals, and W_0_ is the initial TP content in hydrogel beads (mg). 

### 4.7. TP Release Kinetics

The release kinetics of TP from CMC-SA-MXene@TP hydrogel beads were described using mathematical pharmacokinetic models fitted to experimental data [64].

(3) $$Q_{t}=\frac{M_{t}}{M_{\infty }}\times 100\%$$

The zero-order model equation:


(4) $$Q_{t}=k_{0}t$$

The first-order model equation:


(5) $$Q_{t}=1-\exp(-k_{1}t)$$

The Higuchi model equation:

(6) Qt=kHt12

The Ritger–Peppas model equation:

(7) $$Q_{t}=k_{p}t^{n}$$

where *M_t_* is the cumulative released drug amount at time *t*, *M_∞_* is the peak releasable amount, and parameters *k*, *Q_t_*, *n*, and *t* correspond to the kinetic constant, fractional release, diffusion exponent, and elapsed time, respectively. 

### 4.8. DPPH Free Radical Scavenging Activity

The study assessed the DPPH radical scavenging activity of hydrogel beads as per the method of Feng et al. [65], first dissolving DPPH in ethanol to prepare a 0.5 mmol/L DPPH reaction solution and then mixing 2 mL sample solution (0.1 mg/mL) with 1 mL DPPH solution for half an hour of incubation at 25 °C in darkness. We measured the absorbance of each solution at 517 nm, taking pure ethanol as a blank control. Equation (8) below interprets the DPPH radical scavenging activity:

(8) $$DPPH\left(\%\right)=\left(1-\frac{A_{t}}{A_{0}}\right)\times 100\%$$

where *A_t_* and *A*_0_ are the absorbance of the samples and the control group, respectively.

### 4.9. Antibacterial Activity

The study applied *E. coli* and *S. aureus* to evaluate the antibacterial activity of varying hydrogel beads, following the previously reported method [66]. After three successive subcultures, bacterial suspensions were evenly spread onto beef extract-peptone medium that underwent half an hour of high-pressure sterilization (121 °C). Then, the prepared samples received 24 h of incubation on the inoculated medium at 37 °C, with the inhibition zone measured by the agar well diffusion method.

### 4.10. Cell Cytocompatibility and Toxicity

The MTT assay evaluated the cytotoxicity of pulverized CMC-SA-MXene@TP hydrogel beads against HL-7702 and HepG2 [67,68]. Cells seeded in 96-well plates received 24 h of culture in 100 μL of DMEM with 10% FBS and 1% penicillin/streptomycin in specified conditions (37 °C, 5% CO_2_ atmosphere). Then, TP, CMC, MXene, and CMC-SA-MXene@TP hydrogel beads at varying concentrations (0, 5, 10, 25, 50, and 100 μg/mL) were added to the culture medium for an additional 24 h. After PBS wash, cells were replenished with 100 μL of fresh DMEM. Each well was added with MTT solution (10 μL, 5 mg/mL) and allowed for 4 h of incubation, with the resulting purple formazan crystals undergoing solubilization using 100 μL SDS dissolved in a water–DMSO mixture. The NIR treatment group received 6 h of incubation using CMC-SA-MXene@TP, followed by 5 min of irradiation with an 808 nm laser (1.5 W/cm^2^). Absorbance (optical density, OD) was examined via a microplate reader at 570 nm for cell viability quantification. Each experiment was conducted in quintuplicate. Equation (9) below interprets the cell viability (Cᵥ):

(9) $$C_{v}=\frac{OD_{sample}-OD_{blank}}{OD_{control}-OD_{blank}}\times 100\%$$

CLSM captured the fluorescence micrographs after cells from varying treatment groups underwent live/dead staining.

### 4.11. Statistical Analysis

All experiments were repeated in triplicate, with results in the format of the mean ± standard deviation (SD). Statistical analyses relied on Origin (Version 8.5) software. *p* < 0.05 denoted statistical significance.

### 4.12. Comparative Application of SA/MXene Hybrid Systems in the Biomedical Field

Table 3 presents a comparison of parameters related to the application of SA/MXene hybrid systems in the biomedical field between this study and other relevant research. Comparative parameters analysis reveals that these hydrogel microspheres exhibit outstanding pH and near-infrared responsiveness, biocompatibility, drug loading efficiency, antioxidant capacity, and antibacterial activity, making them highly promising for future applications in oral drug delivery systems.

## Figures and Tables

**Table 1:** Loading capacity of CMC-SA@MXene@TP hydrogel beads.

Samples	Additions of TP(g)	Loading Capacity (LC%)
CMC-SA-MXene@TP-1	0.25	15.68 ± 0.72 ^a^
CMC-SA-MXene@TP-2	0.50	34.43 ± 0.87 ^b^
CMC-SA-MXene@TP-3	0.75	37.02 ± 0.98 ^c^

The data are expressed as means ± SD from three independent experiments. Different superscript letters mean significant differences in the same column (*p* < 0.05).

**Table 2:** Zone diameter of different samples.

Samples	*E. coli*	*S. aureus*
CMC	7.25 ± 0.32 ^a^	7.68 ± 0.41 ^b^
MXene	11.03 ± 0.58 ^c^	12.72 ± 0.78 ^c^
TP	26.58 ± 1.42 ^d^	31.33 ± 1.74 ^e^
CMC-SA-MXene@TP	15.31 ± 0.86 ^c^	19.29 ± 0.93 ^d^

Values are means ± SD of triplicate. Different superscript letters mean significant differences in the same column (*p* < 0.05).

**Table 3:** Comparison of parameters related to the application of SA/MXene hybrid systems in the biomedical field.

Samples	Model Drug	Drug Loading Capacity (%)	Antibacterial Properties	Experimental Cells	Cytotoxicity (IC50 Concentration)	Drug Release Mechanism	References
Sodium alginate/MXene/nano-hydroxyapatite hydrogel (SA/MXene/nHAp)	Dorubixin	NR	*E. coli* *S. aureus*	MC3T3-E1	NR	NR	[69]
Ciprofloxacin (CIP)-loaded MXene/sodium alginate (SA) hydrogel (CIP-MX@Gel)	Ciprofloxacin	NR	*E. coli* *S. aureus*	HDF	NR	NR	[70]
Alginate-based hydrogel microspheres containing lysozyme and MXene (i-Lyso@Alg)	Lysozyme	NR	*S. aureus*	NIH 3T3	NR	NR	[71]
Cross-linker-independent oral in situ hydrogel (CCS/CMC-Na)	Chlorogenic acid	NR	*Lactobacillus*, *Muribaculum intestinale*, *Lactobacillus gasseri*, *Bacteroides_sp_ HF-5287*, *Phocaeicola vulgatus*, *E. coli.*	Caco-2	NR	NR	[72]
Carboxymethyl Chitosan/Sodium Alginate/MXene Hydrogel beads (CMC-SA-MXene@TP)	TP	37.02%	*E. coli* *S. aureus*	HL-7702 HepG2	26.51 μg/mL	Ritger–Peppas model	This study

## Data Availability

The original contributions presented in this study are included in the article and the Supplementary Materials.

## Supplementary Materials

The following supporting information are available at https://www.mdpi.com/article/10.3390/gels11121009/s1. Figure S1: Fitted curves of experimental TP release data from CMS-SA@MXene@TP hydrogel beads by various release models at pH of 1.8, 6.8, and 7.8 in the absent of NIR; Figure S2: Fitted curves of experimental TP release data from CMS-SA@MXene@TP hydrogel beads by various release models at pH of 1.8, 6.8, and 7.8 in the present of NIR. Table S1: Parameters of drug release models of CMS-SA@MXene@TP hydrogel beads at pH of 1.8, 6.8, and 7.8 in the conditions of presence or absence.
