# Supplementary material for: Smart pH/Near-Infrared Light-Responsive Carboxymethyl Chitosan/Sodium Alginate/MXene Hydrogel Beads for Targeted Tea Polyphenols Delivery
Source: Gels. 2025 Dec 16;11(12):1009. doi: 10.3390/gels11121009 (PMC12733298; doi:10.3390/gels11121009)
Supplement: Supplementary file 1 [file gels-11-01009-s001.zip › gels-4035550-supplementary.pdf]

Supporting information

Smart pH/Near-infrared Light-responsive Carboxymethyl Chitosan/Sodium Alginate/MXene Hydrogel beads for Targeted Tea Polyphenols Delivery

Kun Fang[1,2]*, Pei Li[3], Hanbing Wang [1,2], Xiangrui Huang[1,2], Yihan Li [1,2], Bo Luo [1,2]*

[1] College of Tea and Food Science, Dabie Mountain Laboratory, Xinyang Normal University, Xinyang, Henan 464000, China
[2] Henan Key Laboratory of Tea Plant Biology, Xinyang, Henan 464000, China
[3] Huaihe Campus Administrative Committee, Xinyang Normal University, Xinyang, Henan 464000, China
* Corresponding authors, E-mail address: fangkun@xynu.edu.cn (Kun Fang); luobo2011@163.com (Bo Luo)

Address: 237 South Lake Road, Shihe District, Xinyang City, Henan Province, China

Table S1. Parameters of release models of CMS-SA@MXene@TP hydrogel beads at pH of 1.8, 6.8, and 7.8 in the conditions of presence or absence.

NIR	pH	Mathematical models								
		Zero order		Frist order		Higuchi		Ritger-Peppas		
		k_0	R^2	k_1	R^2	k_H	R^2	k_p	n	R^2
Absent	**1.8**	0.4964	0.8425	0.1607	0.9603	2.383	0.9768	2.686	0.4597	0.9811
	**6.8**	2.078	0.9050	0.3279	0.9346	9.979	0.907	8.743	0.5376	0.9903
	**7.8**	2.658	0.9125	0.1606	0.9479	13.07	0.9811	10.55	0.5609	0.9817
Present	**1.8**	0.7072	0.8600	0.1884	0.9715	3.824	0.9744	3.996	0.4818	0.9756
	**6.8**	2.540	0.8466	0.1953	0.9424	14.05	0.9807	15.92	0.4624	0.9840
	**7.8**	3.479	0.8918	0.2243	0.9263	17.63	0.9861	18.67	0.4815	0.9869

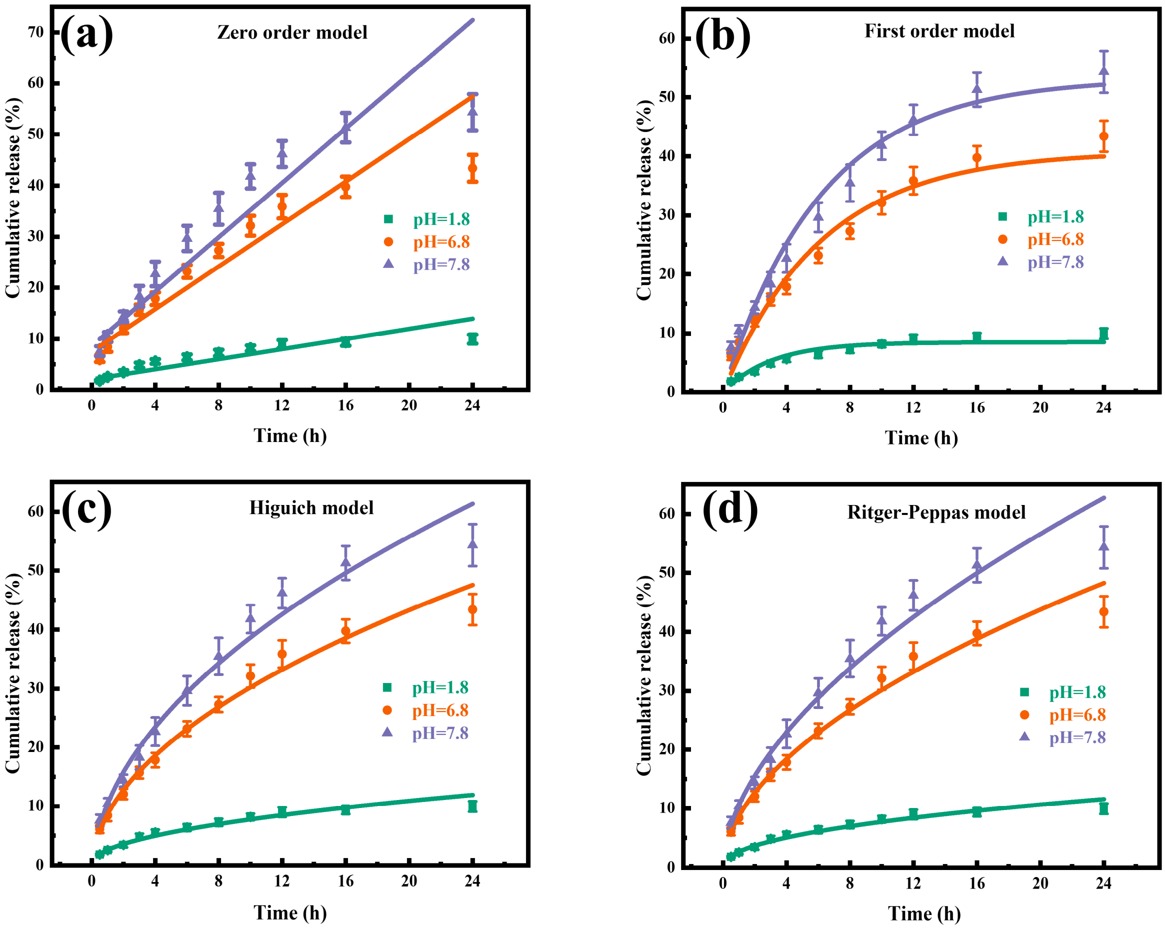

Figure S1. Fitted curves of experimental TP release data from CMS-SA@MXene@TP hydrogel beads by various release models at pH of 1.8, 6.8, and 7.8 in the absent of NIR.

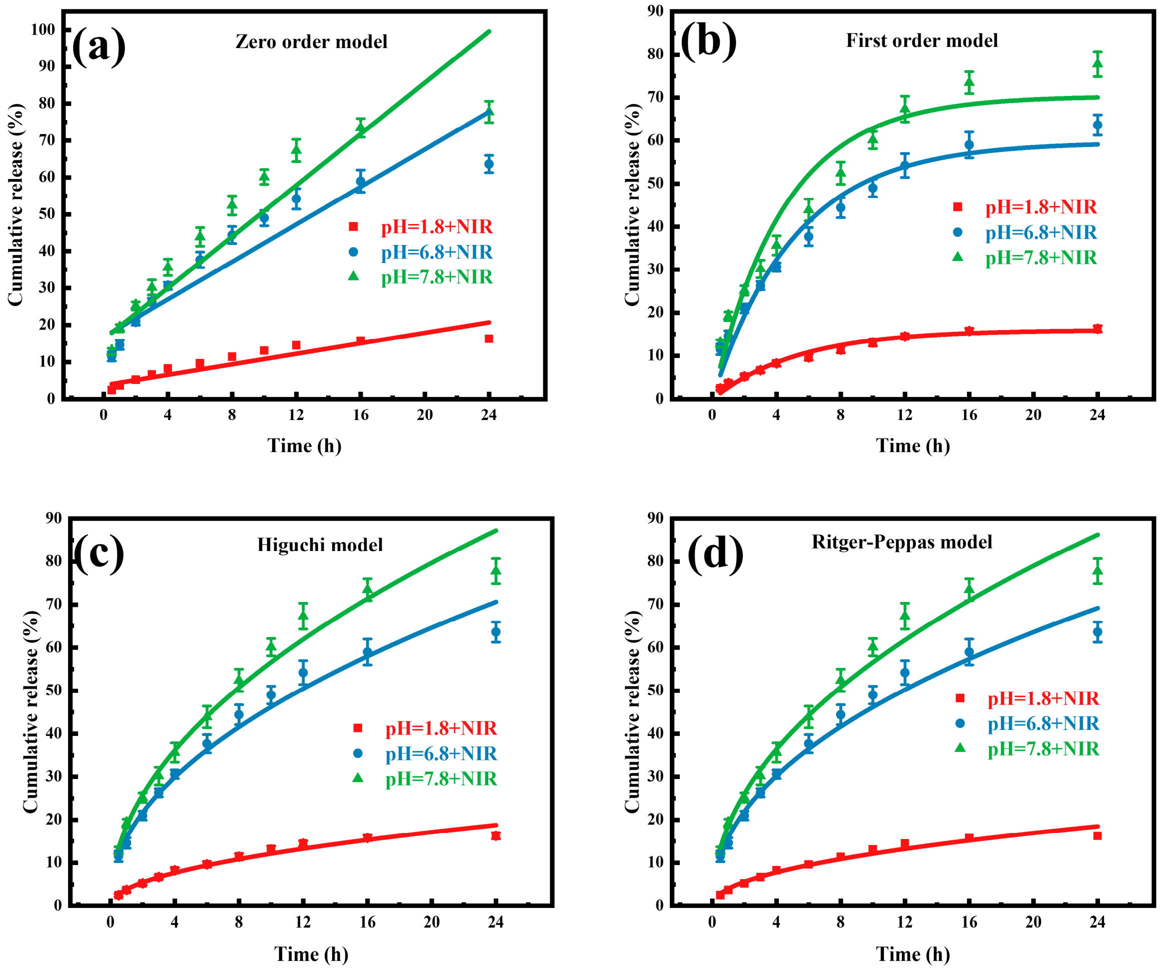

Figure S2. Fitted curves of experimental TP release data from CMS-SA@MXene@TP hydrogel beads by various release models at pH of 1.8, 6.8, and 7.8 in the present of NIR.
